# Dynamic star allele definitions in Pharmacogenomics: impact on diplotype calls, Phenotype predictions and statin therapy recommendations

**DOI:** 10.3389/fphar.2025.1584658

**Published:** 2025-05-23

**Authors:** Sven van der Maas, Simon Denil, Brigitte Maes, Gökhan Ertaylan, Pieter-Jan Volders

**Affiliations:** ^1^ Limburg Clinical Research Center (LCRC), UHasselt, Diepenbeek, Belgium; ^2^ Unit Health, Environmental Intelligence, Flemish Institute for Technological Research (VITO), Mol, Belgium; ^3^ Laboratory of Molecular Diagnostics, Jessa Hospital, Hasselt, Belgium; ^4^ UHasselt, BIOMED, Diepenbeek, Belgium

**Keywords:** pharmacogenomics, pharmvar, star allele, diplotype, GeT-RM, statin recommendation

## Abstract

**Introduction:**

Pharmacogenomics investigates the impact of genetic variation on drug metabolism, enabling personalized medicine through optimized drug selection and dosing. This study examines the effect of the dynamic star allele nomenclature system on diplotypes and therapeutic recommendations using the GeT-RM dataset while also presenting a revised version to address outdated diplotypes.

**Materials and methods:**

PharmVar data up to version 6.2 were downloaded to analyze the evolution of the star allele nomenclature system. FASTQ files from 70 samples of the GeT-RM project were downloaded and aligned to GRCh38, followed by star allele calling using Aldy, PyPGx, and StellarPGx. Diplotypes of the samples were updated based on predefined criteria. Phenotype predictions and therapeutic recommendations were inferred using the PyPGx core API, with CPIC guidelines applied for statin-phenotype combinations.

**Results:**

We reevaluated 1400 diplotypes across 20 pharmacogenes in 70 samples from the GeT-RM dataset using three star allele callers: Aldy, PyPGx, and StellarPGx. Our analysis revealed inconsistencies in 15 of 20 pharmacogenes, with 272 (19.4%) diplotypes being outdated. *SLCO1B1* showed the highest number of discrepant calls, impacting statin dosing recommendations for NA19226.

**Discussion:**

Our findings demonstrate that outdated allele definitions can alter therapeutic recommendations, emphasizing the need for standardized approaches including mandatory PharmVar version disclosure, implementation of cross-tool validations, and incorporation of confidence metrics for star allele calling tools to ensure reliable pharmacogenomic testing.

## 1 Introduction

Pharmacogenomics (PGx) investigates the impact of genetic variation on an individual’s ability to metabolize drugs. PGx plays a central role in enabling the advancement of personalized medicine in healthcare systems through optimizing drug selection and dosage (Singh, 2020). Recent studies have shown that genotype-guided treatment can effectively reduce the incidence of adverse drug events by up to 30% (Swen et al., 2023).

With a growing understanding of the genetic basis behind variable drug metabolism, curation and annotation of pharmacogenetic data became essential (Nebert, 2000). To address this, the star allele nomenclature system was developed, providing a standardized approach for describing pharmacogenetic variants (Robarge et al., 2007). This system allows for the classification of alleles based on their functional impact on enzyme activity. Consequently, an individual’s combination of two star alleles, typically called diplotype, can be translated into a phenotype reflecting the metabolization rate. Based on ones metabolization rate, individuals are generally categorized into four groups: poor metabolizer, normal metabolizer, rapid metabolizer, and ultra-rapid metabolizer (Zanger et al., 2004). These can then be used in evidence-based guidelines for therapeutic recommendations, such as those formulated by the Clinical Pharmacogenomics Implementation Consortium (CPIC) and the Dutch Pharmacogenetics Working Group (DPWG) (Relling and Klein, 2011; Swen et al., 2008).

Currently, the Pharmacogene Variation Consortium (PharmVar) serves as a cornerstone of pharmacogenetic research, as it curates the star allele nomenclature system for 15 major pharmacogenes, including several Cytochrome P450 (*CYP*) genes (Gaedigk et al., 2021). The definitions of star alleles are regularly updated as new star alleles are identified and experimentally validated. Additionally, the PharmVar database incorporates functional annotations for star alleles and their impact on protein activity. PharmVar collaborates closely with the Pharmacogenomics Knowledgebase (PharmGKB) and CPIC (Thorn et al., 2013) to ensure consistency and appropriate standardization. PharmVar focuses on standardizing pharmacogenetic variants, while PharmGKB and CPIC complement this by providing guidelines and more detailed clinical information about the pharmacogenetic variants (Gaedigk et al., 2020).

Several tools exist to determine an individual’s combination of star alleles (Twesigomwe et al., 2021; Hari et al., 2023; Lee et al., 2022). These tools typically compare the genetic variants of an individual with a database of annotated variants. As star allele definitions are continuously evolving, it is essential that the annotation used in the tool is regularly updated and synchronized with the PharmVar definitions.

The implications of the dynamic nature of the star allele nomenclature system on the translation of diplotypes into therapeutic recommendations are poorly understood. Therefore, this study examines the impact of the dynamics of this nomenclature system on the diplotype and therapeutic recommendations of samples from the Genetic Testing Reference Materials Coordination Program (GeT-RM) (Pratt et al., 2016). The GeT-RM dataset offers robust, experimentally validated reference materials for benchmarking, making it a crucial resource for the development and validation of star allele calling tools and genotyping assays. Since star alleles are updated continuously, several diplotypes in the GeT-RM dataset are outdated and require reassessment. In addition to evaluating the impact on therapeutic recommendations of outdated star alleles, we also present an extension of the GeT-RM dataset with updated diplotype calls for 69 out of 70 samples.

## 2 Materials and methods

### 2.1 Star allele calling

FASTQ files of 70 samples of the GET-RM project were downloaded from the European Nucleotide Archive with study accession id PRJEB19931. These FASTQ files were aligned to GRCh38 using the BWA-MEM algorithm (version 0.7.17) (Li and Durbin, 2009), followed by sorting and indexing with Samtools (version 1.18) (Li et al., 2009). Duplicates were marked using Picard Toolkit (version 3.1.1) (Picard Toolkit, 2019). Pre-processed BAM files were then called for star alleles using Aldy (version 4.5), PyPGx (version 0.25.0) and StellarPGx (version 1.2.7) (Hari et al., 2023; Lee et al., 2022; Twesigomwe et al., 2021).

### 2.2 PharmVar analysis

All available PharmVar versions up to 6.2 were downloaded from the PharmVar website (https://www.pharmvar.org/download). Sub alleles were removed and core star alleles were counted for each gene. *DPYD* was excluded from this analysis because *DPYD* variants should be described using Human Genome Variation Society nomenclature rather than legacy star alleles (Pratt et al., 2024).

### 2.3 Revision of diplotypes in GeT-RM dataset

The consolidated consensus PGx diplotypes were downloaded from the Center for Disease Control and Prevention web page (https://www.cdc.gov/lab-quality/php/get-rm/reference-materials.html). A set of criteria was defined to update the diplotypes of the 70 reference samples from the GeT-RM dataset. The criteria for updating diplotypes included: (1) When all diplotype callers identify the same diplotype that differs from the original GeT-RM call, the consensus call was accepted. (2) When the majority of callers identified an allele that could be verified through other sources such as other benchmarks or manual inspection, this call was accepted. (3) In cases where the majority of tools called an allele that extended the ground truth allele (e.g., *5 changed to *2 if *5 includes all core variants of *2), the update was applied. For wild-type alleles, changes were made when one or more callers identified a recently added allele, provided all other callers either called the wild-type allele (suggesting outdated allele databases in those callers) or the same new allele. (4) Obsolete alleles no longer present in PharmVar were removed. and (5) In cases of ambiguous calls where no consensus emerged among callers, the original experimentally observed GeT-RM allele was retained if the calling tools contained this allele in their databases. Based on these criteria, 70 samples from the GeT-RM dataset were subjected to review and updated if the criteria were met. The updated diplotypes and their respective criteria are provided in Supplementary Table S1. *UGT2B7*, *UGT2B15*, *UGT2B17*, *SLCO2B1*, *SLC15A22*, and *SLC22A2* were not included in the analysis due to limited implementation in current star allele calling algorithms, which impeded cross-tool validation. *CYP2E1* was not included in the reevaluation due to insufficient consistency across star allele callers.

### 2.4 Phenotype prediction and therapeutic recommendations

Phenotypes for each diplotype were predicted using the PyPGx core API (Lee et al., 2022). Recommendations for each statin-*SLCO1B1*-phenotype combination were used from the CPIC guidelines and programmatically accessed using the PyPGx core API. For the recommendations of fluvastatin and rosuvastatin, *CYP2C9* and *ABCG2* were assumed to have a normal metabolizer profile.

## 3 Results

### 3.1 The star allele content of PharmVar is highly dynamic over time

**FIGURE 1:**
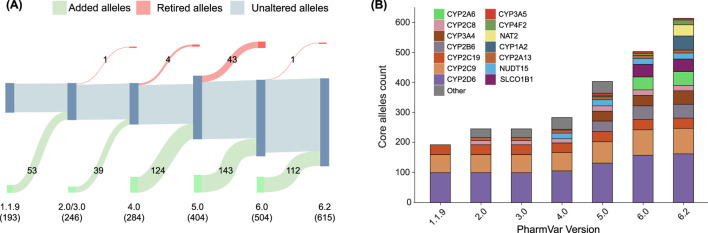
Evolution of the number of core star alleles throughout PharmVar versions. **(A)** Sankey diagram detailing the number of added (green), retired (red), and unaltered (gray) alleles in each version of the PharmVar database. Numbers in parentheses indicate the total allele count for each version. **(B)** Bar plot showing the number of alleles per gene in each major version since release.

We examined the number of updates to core star alleles in the PharmVar database from the earliest accessible version (1.1.9) to version 6.2. During this period, 471 core alleles were added, while 49 core alleles were redefined or removed (Figure 1A). This increase in core alleles, accelerated by the emergence of high-throughput sequencing technologies, reflects the advancements of pharmacogenetic research, with new star alleles being continuously discovered, characterized, and curated (Russell et al., 2021).

Figure 1B illustrates the number of major star alleles across different pharmacogenes throughout these versions. Between 1.1.9.2 and 2.0, several genes from the *CYP* family were added, resulting in an addition of 53 major alleles. A major version release of 3.0 did not include any additional star alleles. However, this version included an update of the nomenclature system. One major allele was redefined/removed from *CYP2C19* between version 3.0 and 4.0. Moreover, 39 major alleles were added in total from version 3.0 to 4.0. Between version 4.0 and 5.0, the star allele definitions of *CYP2B6* were incorporated into the database. Additionally, *CYP2A6* was added to the database in version 5.2. Version 6.0 marked a significant revision of the database, characterized by the retirement of several *CYP* genes and the elimination of previously retired genes from the active database. In version 6.1, *NAT2* was incorporated in the PharmVar database, which was originally curated by the Arylamine N-acetyltransferase Gene Nomenclature Committee (Hein et al., 2013). Lastly, the most recent version (6.2) also provides a newly curated nomenclature for *CYP1A2*. These iterative updates to the PharmVar database highlight the dynamic nature of this repository and underscore the importance of maintaining up-to-date star allele definitions in clinical practice, research settings, and reference materials.

### 3.2 Reevaluation of GeT-RM dataset using star allele callers

**FIGURE 2:**
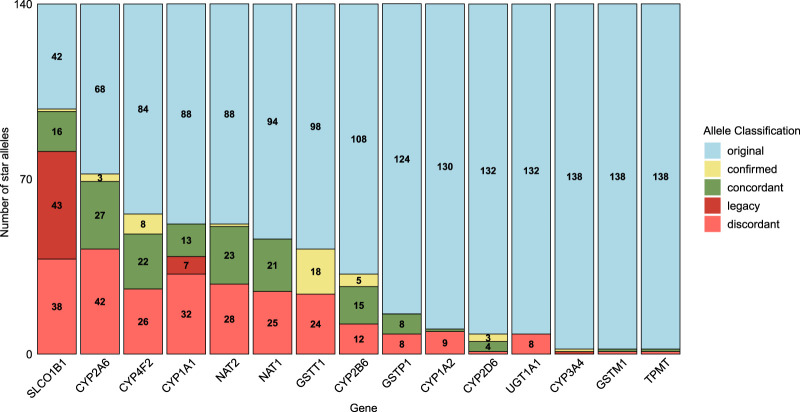
The number of star alleles classified as discordant, legacy, concordant, confirmed, or original in the reevaluated dataset compared to the GeT-RM. Original alleles (light blue) remained unchanged and up to date; confirmed alleles (yellow) were initially tentative in GeT-RM, but validated by multiple tools in this study. Concordant alleles (green) showed agreement between the original dataset and star allele callers, whereas legacy alleles (red) were outdated, and discordant alleles did not align with the tools.

Based on the diplotype calls of Aldy, PyPGx, and StellarPGx, we reevaluated 1400 diplotypes in 20 pharmacogenes of 70 whole genome sequencing (WGS) samples from GeT-RM. In total, 272 diplotypes (19.4%) were updated according to a predefined set of criteria (Supplementary Table S1). 339 (12.1%) star alleles investigated in the GeT-RM dataset, were identified as outdated or inconsistent with current star allele definitions and required revision. To examine the root cause of these discrepancies, the revised alleles were classified in five groups: original, confirmed, concordant, legacy and discordant star alleles. Original and concordant alleles remain unchanged between the original dataset and our revised version. Confirmed alleles were initially tentative, with insufficient experimental evidence in the original GeT-RM dataset, but were validated by multiple star allele callers in our reanalysis. Legacy star alleles use outdated nomenclature systems, and discordant alleles do not align with the results of Aldy, PyPGx and StellarPGx. 15 out of 20 pharmacogenes show at least one inconsistent diplotype (Figure 2). The extent of these inconsistencies varies considerably across genes, with *SLCO1B1* demonstrating the highest number of discrepant calls, attributed by 43 legacy calls and 38 discordant calls. One tentative call for sample NA21781 was confirmed (*5/*15) in *SLCO1B1*, and 42 alleles remained unchanged.

Next to classifying the star alleles, we provide a reevaluated version of the GeT-RM dataset and found star alleles not previously reported by GeT-RM (see Table 1). We identified 33 additional star alleles using Aldy, PyPGx, and StellarPGx. The majority of these star alleles were previously reported by other studies (Hari et al., 2023; Lee et al., 2019; Liu et al., 2023). However, eight star alleles were not reported yet. These alleles were not included in the original gene panels used for GeT-RM characterization and could have remained undetected due to their relatively recent incorporation into star allele calling algorithms (Pratt et al., 2016). *SLCO1B1* shows the highest number of previously unreported star alleles by GeT-RM, while CYP2A6*46 has the highest allele frequency (17.9%) of the unreported star alleles. Several newly defined star alleles were found in the GeT-RM dataset for *NAT2*. These include NAT*38, NAT*39, NAT*44 and NAT*47 with respective allele frequencies of 0.71%, 1.43%, 0.71% and 0.71%. Other newly reported alleles are CYP4F2*5, CYP4F2*6 and CYP4F2*7.

**TABLE 1:** Star alleles identified in this study and not previously reported by GeT-RM (n = 70).

Gene	Star allele	Frequency (%)	Previous reports
*CYP1A1*	*2A	9.29	H, L
	*2B	4.29	H, L
	*11	0.71	H, L
	*13	7.14	H, L
*CYP1A2*	*14	0.71	H, L
*CYP2A6*	*1x2	0.71	H
	*7	1.43	H, L
	*15	0.71	H, L
	*19	1.43	H, L
	*23	0.71	H, L
	*24	1.43	H, L
	*35	2.14	H, L
	*46	17.9	—
*CYP2B6*	*17	0.71	H
	*23	0.71	H, L, P
*CYP2D6*	*106	0.71	H, P
*CYP4F2*	*5	7.14	—
	*6	3.57	—
	*7	0.71	—
*NAT1*	*10	15.0	L
*NAT2*	*38	0.71	—
	*39	1.43	—
	*44	0.71	—
	*47	0.71	—
*SLCO1B1*	*20	2.86	H, L, P
	*27	1.43	H, L, P
	*31	0.71	H, L, P
	*39	0.71	H, P
	*41	1.43	H, P
	*42	0.71	H, P
	*43	0.71	H, P
	*44	0.71	H, P
	*46	0.71	H, P

Dashes (−) indicate no previous reports in the literature on this dataset. H: reported by Hari et al. (2023); L: reported by Lee et al. (2019); P: reported by Liu et al. (2023).

### 3.3 Outdated alleles can have an important impact on therapeutic dosage recommendations

Given its high number of discordant calls, we focused on *SLCO1B1* as a case study to evaluate the clinical implications of updated allele definitions. *SLCO1B1* plays a crucial role in statin pharmacokinetics, and changes in its activity can significantly alter therapeutic recommendations (Romaine et al., 2010). Alterations in metabolizer class were observed in three samples (4.3%) for *SLCO1B1*, not taking into account indeterminate phenotypes (Supplementary Figure S1). Table 2 summarizes the impact of the inconsistency of the *SLCO1B1* call in NA19226 between GeT-RM and our study on statin dosage recommendations. While the original GeT-RM diplotype (*1/*1) indicated normal function and suggested standard dosing for all statins based on disease-specific guidelines, our reevaluation identified a SLCO1B1*31/*37 diplotype, indicating a decreased function. This leads to more cautious dosing recommendations: alternative statins should be considered for simvastatin and lovastatin (with dose limits of <20 mg/day if therapy is warranted), increased monitoring is needed for rosuvastatin and pravastatin (especially at doses >20 mg and >40 mg per day respectively), specific dose limitations apply for atorvastatin ( ≤ 40 mg starting dose) and pitavastatin ( ≤ 2 mg starting dose), and careful monitoring is recommended for fluvastatin at doses >40 mg per day. For both atorvastatin and pitavastatin, the guidelines suggest considering combination therapy with non-statin medications if higher doses are needed for desired efficacy (Cooper-DeHoff et al., 2022).

**TABLE 2:** Therapeutic recommendations based on *SLCO1B1* diplotype of NA19226 from the CPIC guidelines (Relling and Klein, 2011).

GeT-RM		Updated version
Genotype and phenotype information		
Diplotype	*1/*1	*31/*37
Phenotype	Normal function	Decreased function
Therapeutic recommendations by statin		
Statin	Original recommendation	Revised recommendation
Simvastatin	Prescribe desired starting dose and adjust doses based on disease-specific guidelines	Prescribe an alternative statin depending on the desired potency. If simvastatin therapy is warranted, limit dose to < 20 mg/day
Rosuvastatin	Prescribe desired starting dose and adjust doses of rosuvastatin based on disease-specific and specific population guidelines	Prescribe desired starting dose and adjust doses of rosuvastatin based on disease-specific and specific population guidelines. Prescriber should be aware of possible increased risk for myopathy especially for doses > 20 mg
Lovastatin	Prescribe desired starting dose and adjust doses based on disease-specific guidelines	Prescribe an alternative statin depending on the desired potency. If lovastatin therapy is warranted, limit dose to ≤ 20 mg/day
Pravastatin	Prescribe desired starting dose and adjust doses based on disease-specific guidelines	Prescribe desired starting dose and adjust doses of pravastatin based on disease-specific guidelines. Prescriber should be aware of possible increased risk for myopathy with pravastatin especially with doses > 40 mg per day
Atorvastatin	Prescribe desired starting dose and adjust doses based on disease-specific guidelines	Prescribe ≤ 40 mg as a starting dose and adjust doses of atorvastatin based on disease-specific guidelines. Prescriber should be aware of possible increased risk for myopathy especially for 40 mg dose. If dose > 40 mg needed for desired efficacy, consider combination therapy (i.e., atorvastatin plus non-statin guideline directed medical therapy)
Fluvastatin	Prescribe desired starting dose and adjust doses of fluvastatin based on disease-specific guidelines	Prescribe desired starting dose and adjust doses of fluvastatin based on disease-specific guidelines. Prescriber should be aware of possible increased risk for myopathy especially for doses > 40 mg per day
Pitavastatin	Prescribe desired starting dose and adjust doses based on disease-specific guidelines	Prescribe ≤ 2 mg as a starting dose and adjust doses of pitavastatin based on disease-specific guidelines. Prescriber should be aware of possible increased risk for myopathy especially for doses > 1 mg. If dose > 2 mg needed for desired efficacy, consider an alternative statin or combination therapy (i.e., pitavastatin plus non-statin guideline directed medical therapy)

## 4 Discussion and conclusion

This study underscores the clinical implications of the dynamic star allele nomenclature system in PGx. Our findings demonstrate that outdated allele definitions can substantially impact diplotype assignments and therapeutic recommendations. This is particularly evident in our analysis of *SLCO1B1*, for which updated calls in the GeT-RM dataset resulted in altered statin dosing recommendations for NA19226. While the size of our dataset limits our ability to observe rare variants with clinical impact, population-scale studies conducted on the UK Biobank cohort have demonstrated that nearly one-quarter (22.9%) of individuals carry genetic variants associated with decreased *SLCO1B1* function (McInnes et al., 2021). Given that statins are among the most prescribed medicines, accurate identification of these variants has substantial population-level implications for preventing statin-induced myopathy through appropriate dosage adjustments (Salami et al., 2017).

These results not only highlight the impact of evolving star allele definitions on therapeutic recommendations but also reveal systemic challenges in pharmacogenomic analyses. The use of pharmacogenomic panel tests or star allele calling tools that rely on outdated allele definitions can potentially lead to suboptimal drug dosing regimes, thereby compromising treatment efficacy and patient safety. The root causes of inconsistencies between star allele calling tools can be attributed to several factors. Hard-coding star allele definitions within tools create technical debt, as updates to more recent PharmVar definitions are not automatically incorporated in the internal databases of these tools. Second, there is often limited transparency regarding the source and version of star allele definitions used in star allele callers, creating a ’black box’ scenario for clinicians interpreting results. Additionally, many star allele calling tools lack quality control metrics indicating the confidence level associated with their diplotype assignments, making it more challenging to assess the reliability of test results. To address these challenges, we propose several recommendations for standardization: (1) pharmacogenetic testing reports should mandatorily disclose the PharmVar version used for interpretation; tools that fail to provide this information should be considered unsuitable for clinical application. (2) Implementation of quality control measures that compare results across multiple calling tools on the same sample could serve as an important safeguard, particularly for pharmacogenes with high clinical impact (PharmGKB level 1A). (3) Incorporate transparent confidence scoring systems for each star allele assignment.

Moreover, we provide a reevaluated version of GeT-RM consisting of 33 additional previously unreported star alleles in GeT-RM. Although we provide a revised version of GeT-RM, we strongly advocate for the further development of updateable reference materials for benchmarking and validation purposes, such as the Star Allele Search database (Gharani et al., 2024). The Star Allele Search database is an essential resource in this context as it offers a periodically updated repository, by synchronizing with the latest version of PharmVar, of publicly available 1000 Genomes biospecimens. As the field of PGx continues to evolve rapidly, maintaining up-to-date resources and tools is crucial for translating genetic knowledge into effective, reliable, and personalized therapeutic strategies.

## Data Availability

The original contributions presented in the study are included in the article/Supplementary Material, further inquiries can be directed to the corresponding authors.
