# Supplementary material for: Dynamic star allele definitions in Pharmacogenomics: impact on diplotype calls, Phenotype predictions and statin therapy recommendations
Source: Front Pharmacol. 2025 May 23;16:1584658. doi: 10.3389/fphar.2025.1584658 (PMC12141247; doi:10.3389/fphar.2025.1584658)
Supplement: Supplementary file 2 [file Image1.pdf]

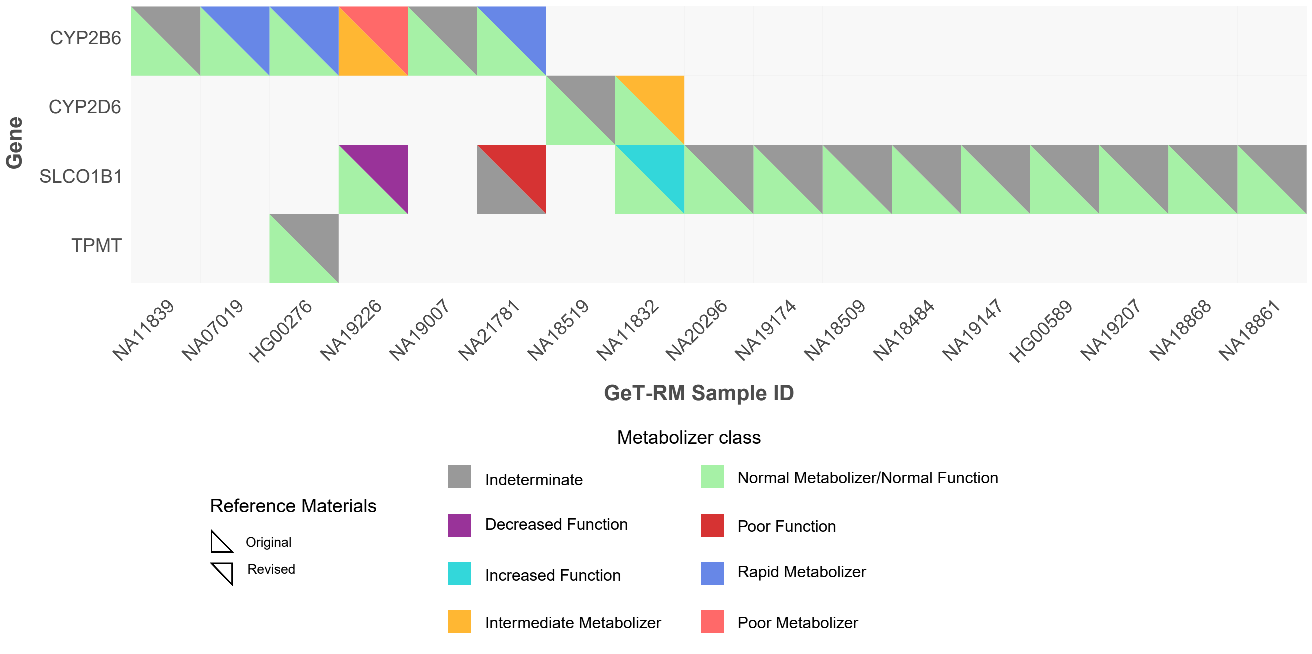

Figure S1. Changes in metabolizer class for GeT-RM samples with updated diplotypes and phenotypes. Genes of which the diplotypes can not be mapped to phenotypes are not included. Bottom-left triangle depicts the metabolizer class in the original callset of GeT-RM, while the top-right triangle represents the revised version.
